# Associations between Periodontal Status and Liver Function in the Japanese Population: A Cross-Sectional Study

**DOI:** 10.3390/jcm12144759

**Published:** 2023-07-18

**Authors:** Toshiya Fujii, Norio Aoyama, Sayuri Kida, Kentaro Taniguchi, Tomomi Yata, Masato Minabe, Motohiro Komaki

**Affiliations:** 1 Department of Periodontology, Kanagawa Dental University, 82 Inaoka-cho, Yokosuka 238-8580, Kanagawa, Japan; t.fujii@kdu.ac.jp (T.F.); kdsyr61@gmail.com (S.K.); k.taniguchi@kdu.ac.jp (K.T.); t.oct.o2m6o@gmail.com (T.Y.); m.komaki@kdu.ac.jp (M.K.); 2 Bunkyou Dori Dental Clinic, 2-4-1 Anagawa, Chiba 263-0024, Chiba, Japan; minabe-m@wk9.so-net.ne.jp

**Keywords:** PISA, periodontal medicine, GGT

## Abstract

A relationship between periodontitis and liver function has been suggested. Indeed, patients with severe periodontal disease have been found to be more prone to liver dysfunction. The periodontal inflammatory surface area (PISA) has been shown to be a useful indicator of periodontal and systemic diseases. However, little information is available regarding whether the PISA is associated with liver function markers, such as gamma-glutamyltransferase (GGT), aspartate aminotransferase (AST), and alanine aminotransferase (ALT). This study aimed to clarify relationship between liver function markers, AST, ALT, and GGT, and PISA level in a cross-sectional study. The subjects were recruited between 2018 and 2021 at the Medical and Dental Collaboration Center of Kanagawa Dental College Hospital. A periodontal clinical examination was performed, and the PISA was calculated. Peripheral blood samples were collected, and serum levels of liver function markers were measured. The levels of liver function markers were examined in different values of PISA. Participants with high PISA scores were more likely to have increased GGT levels while AST and ALT were not changed with PISA. Increased GGT was found in 10.8% and 29.4% (*p* = 0.0056), increased AST in 48.2% and 52.9% (*p* = 0.62), and increased ALT in 35.2% and 47.0% (*p* = 0.20) among <300 mm^2^ and ≧300 mm^2^ PISA groups, respectively. It was found that males with a PISA of 300 mm^2^ or higher had an elevated level of serum GGT. In conclusion, elevated GGT was found in the high PISA group, particularly in males, while AST and ALT did not differ by PISA.

## 1. Introduction

Periodontitis is a disease in which tissue destruction progresses owing to increased chronic inflammation in the periodontal tissue, which causes loss of teeth and oral frailty [1,2]. Bacterial plaques are the main cause of periodontal disease, and various other systemic diseases are known to be associated with periodontal disease [3]. Dysbiosis, an imbalance of bacteria in the oral cavity, is involved in the progression of periodontal disease [4]. Oral microbial dysbiosis can causally link oral disease to extraoral comorbidities via the induction of systemic inflammation or ectopic colonization of oral species in remote tissues [5,6]. The oral bacteria are classified in order of their association with periodontal disease, and the bacteria most associated with periodontal disease are called red complex. The three species in the red complex are *Porphyromonas gingivalis*, *Treponema denticola*, and *Tannerella forsythia* and are considered to be most responsible for severe periodontitis [7]. Periodontopathic bacteria induce persistent immune responses and may affect the whole body if the constituent components of the bacteria in the oral cavity, the bacterial cells themselves, and the inflammatory mediators produced at the lesion flow into the systemic circulation and reach remote organs [8,9,10,11].

Particular attention has been paid to the association between periodontal disease, diabetes, and cardiovascular disease [12,13]. Elevated inflammatory factors in the gingiva of poorly controlled diabetic patients suggest an interactive biological link between periodontitis and diabetes mellitus [10]. In 2017, the European Federation of Periodontology and the International Diabetes Federation published consensus guidelines for physicians, oral health professionals, and patients to improve the early diagnosis, prevention, and management of diabetes and periodontitis [12]. In addition, comprehensive treatment of periodontitis and the rebuilding of healthy periodontal ligaments can reduce inflammation in the body, which may play an important role in the prevention of cardiovascular disease [13]. Among periodontal medicine studies, cardiovascular disease and diabetes mellitus have been frequently reported, and recent reports have also suggested an association between periodontal disease and liver disease [14,15]. Periodontal disease and tooth loss are positively associated with liver diseases including nonalcoholic fatty liver disease (NAFLD), elevated transaminase level, liver cirrhosis and liver cancer [15].

Liver disease is a general term for diseases that cause damage to the liver due to viruses, lifestyle habits, drugs, etc. Altered immune function can alter the pathogenesis of viral hepatitis, autoimmune liver disease, and even hepato-cellular carcinoma [15]. NAFLD more recently referred to as metabolic-associated fatty liver disease, refers to a disease spectrum ranging from hepatic steatosis to nonalcoholic steatohepatitis (NASH), fibrosis, and cirrhosis, associated with hepatic complications and extra-hepatic complications [16]. Many reports have shown that NAFLD is also associated with diabetes [17,18], and liver disease related to diabetes, hepatogenous diabetes, and liver disease occurring coincidentally with diabetes were introduced [17]. Diabetes could be a significant risk factor for progression of the chronic liver disease. In addition, liver function in aging is said to have a significantly reduced regenerative capacity. A report using aging mice has shown that aging causes protein changes and a decrease in regenerative capacity [19]. Moreover, alcohol is broken down and metabolized by the liver. Regular alcohol consumption induces numerous liver changes, including fatty infiltrates, alcoholic hepatitis, and cirrhosis [20]. Thus, liver disease progresses due to a variety of factors.

Some reports suggested a relationship between NAFLD and periodontal disease [21,22,23,24]. Infection with *Aggregatibacter actinomycetemcomitans*, one of the periodontal pathogens, affects NAFLD by altering the gut microbiota and glucose metabolism [22]. Elevated *Porphyromonas gingivalis* in saliva was associated with liver stiffness [23]. An in vivo animal model revealed that infection with periodontopathic bacteria accelerates the progression of NAFLD accompanied by enhanced steatosis [24]. Moreover, the detection of periodontopathic bacteria in the liver may demonstrate that the bacteria have a direct impact on NAFLD [24].

Liver function tests measure the blood markers aspartate aminotransferase (AST), alanine aminotransferase (ALT), and gamma-glutamyl transferase (GGT) [25]. A liver biopsy is one of the reliable liver function tests like blood biomarkers. Using liver biopsies, periodontitis and gingivitis were shown to be associated with liver diseases [26]. Patients with periodontitis had higher CRP levels, while those with gingivitis presented higher GGT levels [26]. AST and ALT are enzymes that perform important functions such as amino acid metabolism and are present in the cells of organs such as the heart, kidneys, and liver [27,28]. GGT is an enzyme present in the bile of digestive fluid that decomposes proteins and fats in the intestine. When alcohol is consumed, the production of GGT is activated and increases [29]. An increasing number of studies have shown that increased GGT levels are associated with NASH [30]. GGT is not only a marker of liver function, but is also associated with various diseases [31]. GGT is an early predictive marker for atherosclerosis, heart failure, arterial stiffness and plaque, gestational diabetes, and various liver diseases, including viral hepatitis, other infectious diseases, and several life-threatening cancers [31]. GGT may be a factor with systemic relevance in addition to liver function.

NAFLD, a precursor to NASH, is recently being under consideration for the accuracy of the disease names. In a 2023 report, 74% of respondents to a survey of 236 panelists from 56 countries felt that the current nomenclature is flawed enough to consider a name change [32]. Metabolically associated fatty liver disease (MASLD) was proposed as an alternative name for NAFLD, and metabolically associated fatty hepatitis (MASH) is being considered as an alternative name for NASH. Although the new nomenclature and diagnostic criteria are widely supported and have the potential to improve patient recognition [32], the terms NAFLD and NASH were used in this study because of their wide usage today.

The periodontal inflamed surface area (PISA) can be used to determine periodontal inflammation based on the clinical attachment level, the periodontal pocket depth, the amount of gingival recession, and the value of bleeding on probing [33]. It is a numerical value that allows a quantitative assessment of periodontal disease. Gingivitis index and bleeding on probing, which are conventional indicators of inflammation in periodontal tissues, are not familiar to medical professionals in the treatment of systemic diseases. As an objective indicator of inflammation in periodontal tissue, PISA can provide information on periodontal conditions to medical professionals in an easily understandable form.

Generally, periodontal treatment begins with a periodontal clinical examination to determine the extent of progression. This is followed by the removal of calculus, plaque, and other exogenous deposits on the teeth, as well as the removal of many other risk factors to promote gingival and root attachment. Removal of calculus is first performed by initial periodontal therapy, which is primarily scaling and root planing (SRP). For teeth that do not improve after reevaluation, periodontal surgery is performed for further improvement. Reevaluation is then repeated to stabilize the periodontal condition. The relationship between periodontal treatment and liver function is gradually becoming clearer. In 2022, Kamata et al. reported that SRP treatment significantly reduced liver enzyme and endotoxin levels in patients with NAFLD and periodontal disease, and was generally well tolerated in these patients [34]. Thus, periodontal treatment may have a positive effect on liver disease.

A link between periodontal disease and liver diseases has been reported; however, there are no reports on the association between the PISA and the liver function markers like GGT, AST and ALT. This study aimed to investigate the relationship between periodontal disease and GGT, AST and ALT levels using the PISA.

## 2. Materials and Methods

### 2.1. Study Population

The subjects were recruited between 2018 and 2021 at the Center for Medical and Dental Cooperation at Kanagawa Dental University Hospital. The participants were required to be at least 20 years old and provide consent to participate. The exclusion criteria were antibiotic intake within the past two months, severe systemic infection, pregnancy, and lactation status. This study was approved by the Ethics Committee of Kanagawa Dental University School of Dentistry (No. 499), and the protocol was performed in accordance with the Declaration of Helsinki (revised in 2013). The study purpose and procedures were explained to each participant. Written informed consent was obtained from all participants prior to their participation.

### 2.2. Clinical Examinations

General information about the participants, such as age and gender, was obtained from interviews and medical records at the initial visit. Subjects had peripheral blood samples taken to measure serum levels of liver function markers such as AST, ALT, and GGT, and hemoglobin A1c, a diabetes marker. Blood glucose levels were measured using a blood glucose meter (Pocket Chem; Arkray, Kyoto, Japan) with a small amount of blood from the subject’s fingertip.

A trained periodontist evaluated the periodontal status of the subjects. Methods included counting the number of remaining teeth, excluding wisdom teeth, and measuring probing pocket depth and probing bleeding at 6 points per tooth for all teeth using a manual probe (PCP-UNC 15, Hu-Friedy, Chicago, IL, USA). Using these periodontal parameters, periodontal epithelial surface area and PISA were calculated using previously reported methods; PISA reflects the surface area of the bleeding pocket epithelium in square millimeters. The surface area of the bleeding pocket epithelium quantifies the amount of inflamed periodontal tissue [33]. Saliva testing was performed using the Sill-Ha (Arkray Inc., Kyoto, Japan) according to the manufacturer’s instructions for use. Briefly describing the method of use, subjects put the solution in their mouths, rinsed their mouths for 10 s, mixed the solution and saliva in their mouths, and collected the mixture by spitting it out into a cup. The collected saliva was applied to a test strip, which was then placed in a dedicated measuring device for testing. From the saliva test, the protein and leukocyte scores in the saliva were calculated to indicate the degree of gingival inflammation.

### 2.3. Statistical Analysis

The report of Leira et al. was referred to when setting the cut-off value of PISA [35]. They showed that the mean PISA values were 34.30 ± 16.48 mm^2^ in the healthy group, 292.74 ± 98.08 mm^2^ in the mild periodontitis group and 645.66 ± 86.29 mm^2^ in the moderate periodontitis group. Thus, the PISA cutoff value for this study was set at 300 mm^2^, referring to the mild group value, and a comparison was performed between high and low PISA groups.

The Shapiro-Wilk test was performed to test the normality of the data distributions. Numerical data are presented as medians and interquartile ranges for skewed distributions. Pearson’s chi-square test was used to compare categorical values. The Wilcoxon test was used to compare differences in values between groups. JMP version 14.2.0 software (SAS Institute Inc., Cary, NC, USA) was used for all statistical analyses. A *p*-value of <0.05 was considered statistically significant.

## 3. Results

The characteristics of the subjects included in this study are shown in Table 1. Numerical data are presented as medians and interquartile ranges for skewed distributions. There were 114 women and 59 men, for a total number of 173. The median age was 69, with a relatively large number of permanent subjects.

Subjects were divided into two groups, according to their PISA scores, and their general health status and oral health values were compared in Table 2. No association was found between age, gender, number of teeth, HbA1c, or blood glucose levels and PISA, while an upward trend of HbA1c was observed in the high PISA group. Probing depth and bleeding on probing were significantly increased in the high PISA group. The liver function markers AST and ALT were not significantly elevated, although there was an increasing trend in the high PISA group; subjects with high PISA had elevated salivary leukocyte and protein scores, and serum GGT levels.

Although the gender difference was not significant, the high PISA group had a high proportion of males (approximately 90%), and GGT test results showed gender differences, so subsequent analyses were conducted separately for males and females.

**Figure 1:**
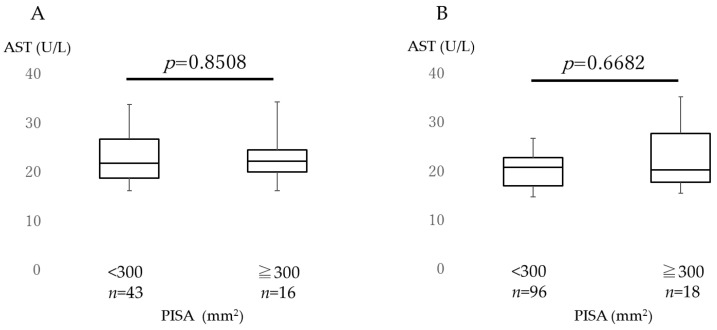
Comparison of aspartate aminotransferase. Aspartate aminotransferase (AST) levels were compared between the high (≥300 mm^2^) and low (<300 mm^2^) periodontal inflamed surface area (PISA) groups. (**A**) Comparison of the males and (**B**) females. The box plots show the medians, 25th, and 75th percentiles as boxes and the 10th and 90th percentiles as whiskers. The Wilcoxon signed-rank test was used.

**Figure 2:**
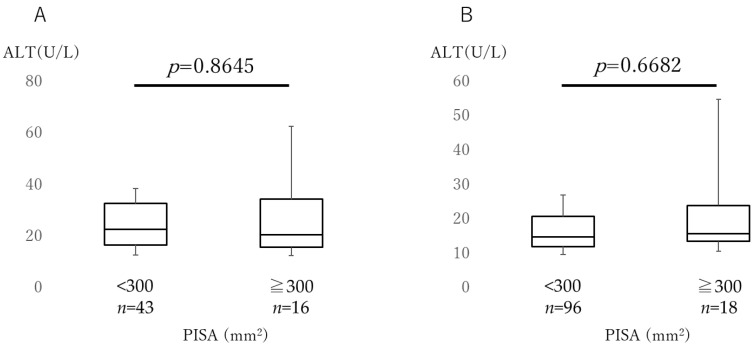
Comparison of alanine aminotransferase. Alanine aminotransferase (ALT) levels were compared between the high (≥300 mm^2^) and low (<300 mm^2^) periodontal inflamed surface area (PISA) groups. (**A**) Comparison of the males and (**B**) females. The box plots show the medians, 25th, and 75th percentiles as boxes and the 10th and 90th percentiles as whiskers. The Wilcoxon signed-rank test was used.

**Figure 3:**
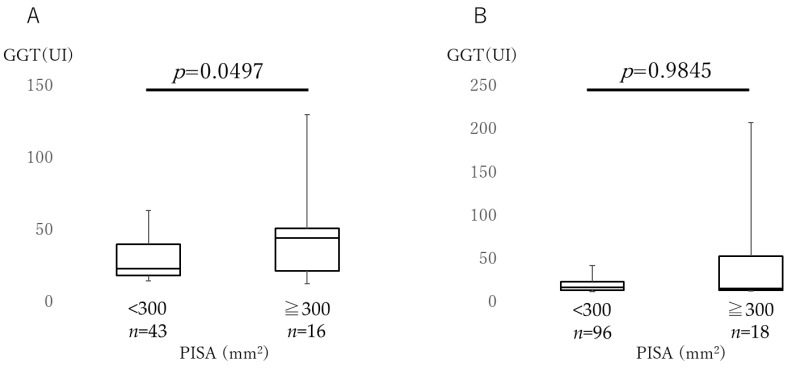
Comparison of gamma-glutamyltransferase. Gamma-glutamyltransferase (GGT) levels were compared between the high (≥300 mm^2^) and low (<300 mm^2^) inflamed periodontal surface area (PISA) groups. (**A**) Comparison of the males and (**B**) females. The box plots show the medians, 25th, and 75th percentiles as boxes and the 10th and 90th percentiles as whiskers. The Wilcoxon signed-rank test was used.

Figure 1 shows the AST levels in both males and females in the low and high PISA groups. Figure 2 shows the ALT levels in both groups. AST and ALT levels were not significantly different between the two PISA groups in both male and female subjects. There were no differences in the AST and ALT levels between the sexes. Figure 3 depicts a comparison of the GGT levels in the low and high PISA groups, considering sex differences. In the high PISA group, the GGT levels were elevated in males (*p* = 0.0497), whereas no significant differences were observed in females (*p* = 0.9845).

**Figure 4:**
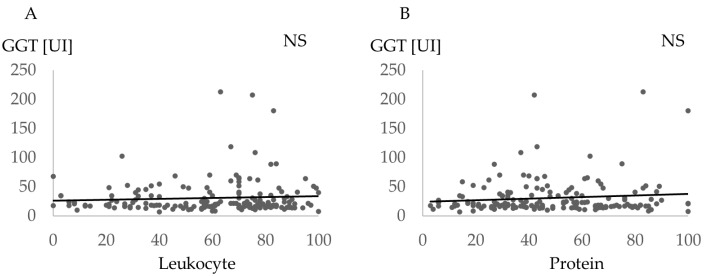
Comparison of gamma-glutamyltransferase and saliva score. Association between gamma-glutamyltransferase (GGT) and the saliva scores such as leukocyte and protein. (**A**) Comparison of the Leukocyte and (**B**) Protein. NS, no statistical correlation.

Figure 4 shows the association between GGT and saliva scores such as leukocyte and protein. No significant correlation was found (leukocyte *p* = 0.36, and protein *p* = 0.16).

## 4. Discussion

In the present study, it was shown that participants with high PISA scores were more likely to have increased GGT levels. The PISA is a new indicator of clinical periodontal conditions and is associated with systemic diseases [36,37]. The current results also showed an increase in probing pocket depth and bleeding on probing in the high PISA group, which confirmed the usefulness of PISA. It has also been reported that the PISA is associated with NASH, liver disease, AST and ALT [38].

The reason why AST and ALT are shown in addition to GGT as markers of liver function in this study is that AST and ALT are the most commonly used scores to evaluate liver function, and some studies have evaluated the relationship to periodontal disease using AST and ALT [39,40]. Recently, GGT has also been used as an indicator of liver function markers, and this study examined the association between GGT and PISA. AST and ALT, which have been widely used in conventional studies, were also shown as secondary markers in this study.

Liver biopsy is an effective method like liver biomarkers in measuring liver function. In a report by Čolak et al., the relationship between periodontal disease and NASH and NAFLD was studied using liver biopsy [26]. The reason why we used biomarkers in our study is that liver biopsy is a rather difficult and specialized method. Biomarkers, on the other hand, are a relatively simple method of collecting and analyzing blood samples. In addition, blood sampling is an acceptable test even for dentists in Japan, so we compared it to liver function biomarkers in this study.

Probing pocket depth and bleeding on probing are conventionally used as periodontal disease indicators. These values have been used in the previous reports which showed an association with liver disease or functions [41,42]. Iwasaki et al. performed an oral health examination and assessed the relationship between periodontal disease status and ultrasound diagnosed NAFLD in Japanese participants [41]. They found that a probing pocket depth of 4 mm or more may be a risk factor for NAFLD [41]. Duseja et al. reported that bleeding on probing was observed to have a higher odds ratio in patients with liver disease, and concluded that patients with liver disease had a higher prevalence of periodontal disease and poorer oral hygiene conditions compared to healthy subjects [42]. While probing pocket depth and bleeding on probing have conventionally been widely used as indices representing the clinical state of periodontal disease, PISA, a novel value of the periodontal clinical condition, can quantify the severity of periodontitis as one value.

As shown in Table 2, the PISA and liver function markers were divided into two groups using cutoff values. The cutoff value for PISA was set at 300 mm^2^ [35]. In this study, fewer subjects had a PISA of 300 mm^2^ or higher, and so this point requires caution in interpreting the results. The cutoff value for GGT was set at 50 IU because a GGT value of 50 IU or higher during a medical checkup in Japan requires reexamination. Because GGT differs between males and females [43], we analyzed the levels of GGT by gender and showed that GGT was increased in the male high PISA group (Figure 3A).

Some previous studies showed the relationship between liver function and periodontal disease. Helenius-Hietala et al. performed a follow-up study with 6165 people without liver disease, resulting in 79 subjects having liver diseases during follow-up [44]. Patients with liver diseases had worse periodontal conditions than the other subjects [44]. Kim et al. indicated that the subjects with high fatty liver index as a predictor of NAFLD had a high prevalence of periodontal disease [45]. GGT levels are elevated in NAFLD, a pre-NASH condition [46]. Many reports have also shown that patients with NAFLD and NASH have poor periodontal conditions [21,47,48]. Although the specific presence or absence of liver disease was not noted in this study, few participants had liver disease. Regarding the relationship between periodontal disease and GGT, Chu et al. reported that *Treponema denticola*, a major periodontal pathogen, increases with GGT levels [49]. GGT levels are significantly elevated in patients with deep periodontal pockets compared to those without periodontal pockets [49]. Our results are consistent with these findings. Periodontal disease is not associated with alcohol intake but with liver function [50]. This suggests that the management and treatment of periodontal disease may improve liver function.

It has been reported that chronic alcohol consumption increases GGT [31,51]. Teschke et al. reported that chronic alcohol consumption leads to an increase in the activity of GGT in serum, which is associated with an enhancement of GGT activity in the liver [51]. Koenig et al. reported that GGT is an early predictive marker for a variety of liver diseases, including atherosclerosis, heart failure, atherosclerosis and plaque, gestational diabetes, viral hepatitis, other infectious diseases, and several life-threatening cancers, in addition to being elevated by alcohol intake [31]. The relationship between alcohol and periodontal disease has been reported by Park et al. indicating that men with higher alcohol intake were more likely to require periodontal treatment regardless of age, socioeconomic factors, general health, and multivariate-adjusted number of toothbrushes per day [52]. The present study lacked information on the subjects’ alcohol intake, which is a major limitation of this study. Data on alcohol intake habits should be taken into account in future studies. 

There have been valid reports on the improvement of liver function status in periodontal disease treatment. A report comparing a group that underwent SRP, a periodontal treatment, with a group that did not undergo SRP discovered a decrease in liver function markers AST, ALT, and GGT in the treatment group [34]. The main objectives of SRP are the removal of subgingival marginal calculus and biofilm deposits to create a biocompatible root surface and reduce inflammatory load. SRP remains important in the treatment of periodontitis and many reports supported improvements of PISA by SRP [53,54,55]. These reports suggest that periodontal treatment may also improve liver function status.

The results of this study showed that GGT levels increased in the high PISA group, whereas the AST and ALT levels did not increase. GGT is an enzyme that degrades proteins in the body, and GGT was reported to be elevated in mice that ingested a large amount of protein [56]. Patients with chronic periodontitis show higher levels of proteins in gingival crevicular fluid [57]. The present results also showed an elevated salivary protein level in the high PISA patients. It is possible that elevated levels of proteins, particularly inflammatory markers, may lead to an increase in GGT in patients with periodontitis.

We also observed an increase in GGT levels in male participants with high PISA scores. Unstable GGT values in females were reported due to changes in female hormones and GGT levels before and after menopause [58]. GGT levels were higher in postmenopausal than premenopausal females [58]. Although many participants in this study were postmenopausal females, their GGT levels were generally low. This may be another reason why no relationship was found between the PISA and GGT levels in females. In addition, males tended to consume more alcohol than females, and their GGT levels were slightly elevated [59]. Some studies reported that alcohol consumption and periodontal disease are unrelated [60,61]; thus, GGT levels may be independently related to periodontal disease.

HbA1c and salivary data such as protein and leukocyte scores were also examined in this study. HbA1c is a score commonly used to evaluate diabetes mellitus and is often reported to be associated with periodontal disease [62,63]. Baeza et al. reported that when periodontal treatment was performed on patients with type 2 diabetes, HbA1c values decreased [62]. The report by Stanko et al. focuses on the bidirectional relationship between diabetes and periodontal disease, with diabetes increasing the risk and severity of periodontitis and periodontal disease exacerbating insulin resistance, which may affect glycemic control [63]. Aoyama et al. showed that elevated HbA1c was found in patients with decreased masticatory function [64], which indicates that diabetes is associated with oral status such as periodontal infection and masticatory function. Saliva is currently being studied for its association with cancer, diabetes, and other systemic diseases [65]. 

In saliva testing, high protein and leukocyte scores were found in the high PISA group. The usefulness of salivary tests was proposed in previous reports [66,67]. Kim et al. [66] studied 10 adult males and females to clarify the measurement principles of salivary tests. It was also evaluated whether salivary proteins and white blood cell counts were associated with the severity of periodontal disease in 13 children with periodontitis and 17 children without periodontitis [67]. Although these studies investigated a relatively small number of subjects, the usefulness of the salivary analyzer was confirmed in this study of 173 subjects. However, saliva scores were not associated with GGT in this study, thus further studies are needed to indicate the usefulness of saliva testing for the assessment of systemic conditions.

There are several limitations to this study. First, the periodontal treatment period of the participants was not specified. In addition, the participants’ periodontal disease was not diagnosed. The subjects in this study were not limited to first-time visitors to the dental clinic. Therefore, the phase of dental treatment varied from subject to subject. Next, subjects’ medication and alcohol consumption were not analyzed in this study due to a lack of data. In particular, alcohol intake not only affects GGT, but should be considered in relation to periodontal disease, thus alcohol is an important parameter for deepening this research topic. Third, the study did not assess the subjects’ liver disease status. Although the specific presence or absence of liver disease was not noted in this study, few participants had liver disease. The presence or symptoms related to liver disease need to be discussed in future studies. Fourth, the sample size was not calculated prior to the study. These issues should be considered when interpreting the results of this study. As for future research prospects, it is important to investigate whether periodontal treatment is effective in improving GGT. In addition, it is necessary to conduct research by taking into consideration the lifestyle background, alcohol intake, smoking status, and periodontal disease diagnosis for each subject.

## 5. Conclusions

In conclusion, elevated GGT, a marker of liver function, was found in the high PISA group. In particular, GGT levels were elevated in males in the high PISA group. In contrast, AST and ALT did not differ between the high and low PISA groups. The relationship between periodontal status and general health should be considered in clinical practice.

## Figures and Tables

**Table 1:** Subject characteristics.

Variables	
*n*	173
Sex [female %]	65.9
Age [years]	69 (60, 76) ^1^
Number of teeth	25 (22, 27) ^1^
PISA [mm^2^]	151.3 (69.8, 270.0) ^1^
HbA1c [%]	5.7 (5.4, 6.0) ^1^
Blood glucose [mg/dL]	111.0 (98.5, 128.5) ^1^
Probing depth [mm]	2.2 (2.1, 2.4) ^1^
Bleeding on probing [%]	18 (9, 33) ^1^
Leukocyte score in saliva	67 (40, 79) ^1^
Protein score in saliva	45 (30, 67) ^1^
AST [U/L]	21 (18, 25) ^1^
ALT [U/L]	17 (13, 25) ^1^
GGT [UI]	20 (16, 35) ^1^

^1^ Data are shown as the median (interquartile range). PISA, periodontal inflamed surface area; HbA1c, hemoglobin A1c; AST, aspartate aminotransferase; ALT, alanine aminotransferase; GGT, gamma-glutamyl transferase.

**Table 2:** Comparison of the general condition according to periodontal status.

	PISA [mm^2^] < 300	PISA [mm^2^] ≧ 300	*p*-Value
Age [years]	69 (50, 75)	71.5 (58, 79)	0.48
Sex [female %]	55.5	10.4	0.07
Number of teeth	25 (22, 27)	26 (22, 28)	0.40
HbA1c [%]	5.6 (5.4, 5.9)	5.8 (5.4, 6.4)	0.24
Blood glucose [mg/dL]	110 (98, 127)	112 (99, 175)	0.25
Probing depth [mm]	2.2 (2.0, 2.4)	3.0 (2.5, 3.3)	<0.0001
Bleeding on probing [%]	14 (8, 23)	47 (35, 62)	<0.0001
Leukocyte score in saliva	59 (35, 77)	77 (70, 89)	<0.0001
Protein score in saliva	42 (28, 65)	58 (43, 83)	0.0001
Increased AST [%]	48.2	52.9	0.62
Increased ALT [%]	35.3	47.0	0.20
Increased GGT [%]	10.8	29.4	0.0056

The subjects were divided into two groups according to the PISA using a cutoff value of 300 mm^2^. Cutoff values were also established for AST, ALT, and GGT; more than 20 U/L of AST, more than 20 U/L of ALT, and more than 50UI GGT were judged as high values. PISA, periodontal inflamed surface area; HbA1c, hemoglobin A1c; AST, aspartate aminotransferase; ALT, alanine aminotransferase; GGT, gamma-glutamyl transferase.

## Data Availability

The data presented in this study are available upon request from the corresponding author.
